# Small Particles, Big Impact: Inorganic Nanotechnology for Glioblastoma

**DOI:** 10.3390/molecules31030565

**Published:** 2026-02-06

**Authors:** Klaudia Dynarowicz, David Aebisher, Jakub Tylutki, Nazarii Kozak, Aleksandra Kawczyk-Krupka, Dorota Bartusik-Aebisher

**Affiliations:** 1 Department of Biochemistry and General Chemistry, Faculty of Medicine, University of Rzeszów, 35-310 Reszów, Poland; kdynarowicz@ur.edu.pl (K.D.); dbartusikaebisher@ur.edu.pl (D.B.-A.); 2 Department of Photomedicine and Physical Chemistry, Faculty of Medicine, University of Rzeszów, 35-310 Reszów, Poland; 3 English Division Science Club, Faculty of Medicine, University of Rzeszów, 35-310 Rzeszów, Poland; jt130173@stud.ur.edu.pl (J.T.); nk130099@stud.ur.edu.pl (N.K.); 4 Department of Internal Diseases, Angiology and Physical Medicine, Center for Laser Diagnostics and Therapy, Faculty of Medical Sciences in Zabrze, Medical University of Silesia, 40-055 Katowice, Poland

**Keywords:** glioblastoma multiforme, blood–brain barrier, inorganic nanoparticles, nanotechnology

## Abstract

Background: Glioblastoma Multiforme (GBM) is one of the most aggressive primary brain tumors, with a median survival of only 15–17 months. Treatment failure is largely driven by the Blood–Brain Barrier (BBB), which restricts the delivery of most conventional therapeutics and shields invasive tumor regions from systemic drugs. Approach: This review highlights recent advances in inorganic nanoparticles designed to cross the BBB and target GBM. These platforms, including silica-, metal-, and carbon-based nanomaterials, enable multimodal applications such as tumor imaging, localized hyperthermia, and selective induction of cancer cell death. Functionalization with targeting ligands or surface modifications further enhances tumor penetration and therapeutic efficacy. Outlook: Despite promising preclinical results, clinical translation requires careful optimization of nanoparticle properties to minimize toxicity and immune clearance. Understanding these challenges provides a roadmap for the development of more effective nanomedicine strategies aimed at improving outcomes for GBM patients.

## 1. Introduction

Glioblastoma Multiforme (GBM) is the most aggressive primary brain tumor, accounting for nearly 50% of all malignant central nervous system (CNS) [1,2] neoplasms and approximately 57% of all gliomas [3]. Despite advances in oncology, GBM remains highly lethal, with a median survival of only 15–17 months and a 2-year survival rate of around 25% [4,5,6,7,8]. The poor prognosis is largely due to several intrinsic features of the tumor, including high heterogeneity, rapid proliferation, invasive growth, and a strong tendency for recurrence, even after initially successful treatment [9,10,11].

Standard treatment for GBM involves maximal safe surgical resection followed by radiotherapy and adjuvant chemotherapy [12,13,14], typically with the alkylating agent temozolomide. While these approaches can temporarily reduce tumor burden, their efficacy is severely limited by the Blood–Brain Barrier (BBB) [15,16,17,18,19], which restricts the delivery of most therapeutic agents to the tumor site. Tumor heterogeneity and differential receptor expression further complicate targeted therapies, including monoclonal antibodies such as bevacizumab or immune checkpoint inhibitors [16,17,18,19]. Moreover, resistance to radiotherapy, chemotherapy, immunotherapy and gene therapy remains a major challenge, resulting in a critical unmet need for more effective treatment strategies [20,21,22].

In recent years, nanotechnology has emerged as a promising avenue to overcome these obstacles. Organic nanoparticles, such as liposomes, polymeric micelles, or dendrimers, offer advantages in terms of biocompatibility and drug encapsulation [23]. However, their clinical performance is often constrained by limited stability, reduced payload capacity, and rapid clearance from circulation. In contrast, inorganic nanoparticles—including silica-, metal-, and carbon-based platforms—possess unique physicochemical properties that enable a broader range of functionalities. These include multimodal imaging, photothermal therapy, radiosensitization, and controlled release of therapeutic agents, while also facilitating BBB penetration through passive accumulation or receptor-mediated transcytosis [24]. The tunable size, surface chemistry, and magnetic or optical responsiveness of inorganic nanomaterials provide a versatile toolkit for addressing the complexity of GBM [25].

This review aims to highlight recent advances in inorganic nanotechnology for GBM treatment, focusing on their capacity to overcome key clinical challenges, including limited drug delivery, tumor heterogeneity, and therapeutic resistance. We discuss strategies for BBB traversal, tumor targeting, and multimodal therapy, while comparing inorganic and organic platforms to emphasize the unique advantages of non-organic nanomaterials. By synthesizing current knowledge, this review provides a framework for understanding how inorganic nanoparticles can improve diagnostic and therapeutic outcomes in GBM, offering new hope for patients.

## 2. Barriers and Nanoparticle-Mediated Drug Delivery Strategies

Distinguished by their capacity to navigate physiological barriers, NPs have ascended as a leading modality in contemporary GBM pharmacotherapy. Their utility lies in precision: NPs enable site-specific targeting and spatiotemporally controlled drug release, thereby maximizing therapeutic indices while minimizing systemic exposure [23].

### 2.1. Bypassing Blood–Brain Barrier and Tumor Targeting

**Figure 1:**
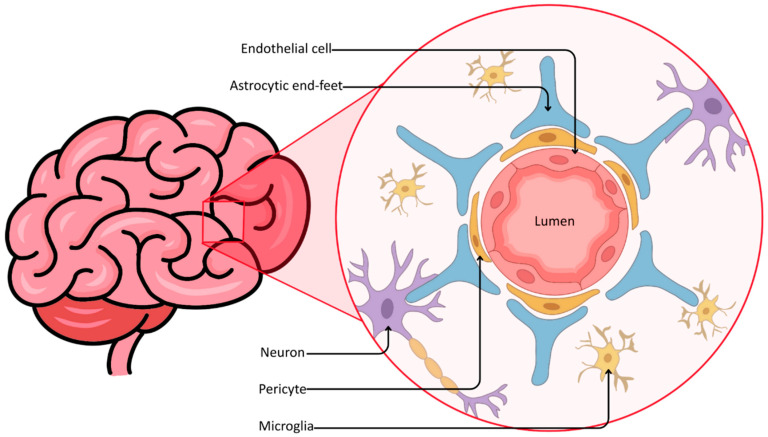
Cellular architecture of the neurovascular unit.

As noted previously, the efficacy of standard pharmacotherapy in GBM is notoriously compromised by the BBB. This barrier derives its complexity from the highly organized neurovascular unit, composed of endothelial cells sealed by tight junctions and supported by pericytes and astrocytic end-feet (Figure 1).

As a result, nearly 98% of small-molecule drugs are excluded from therapeutic access to the brain [24,25]. In contrast, the core of bulky glioblastomas often exhibits a disrupted barrier due to chaotic, fenestrated angiogenesis, forming the so-called blood–brain tumor barrier (BBTB); however, the tumor periphery presents a different challenge [26]. The invasive rim, which defines the infiltrative nature of GBM, harbors migratory glioma cells protected by a largely intact and functional BBB [26,27,28]. It is these shielded cells that invariably drive recurrence. Consequently, a therapeutic paradox emerges: agents capable of killing glioma cells are often unable to reach them at effective concentrations without inducing systemic toxicity.

NPs have emerged as a sophisticated countermeasure, enabling barrier traversal through multiple molecular strategies. To cross the BBB, nanoparticles must either overcome its selectivity via physical modulation or exploit endogenous transport pathways. Their permeation is governed by a complex interplay of size, charge, shape, and surface chemistry, and may be further influenced by external forces.

Receptor-mediated transcytosis (RMT) is a precise mechanism where NPs are surface-functionalized with ligands such as transferrin, lactoferrin, or angiopep-2. These moieties bind specifically to receptors overexpressed on the luminal side of brain endothelial cells, triggering vesicular transport across the barrier [29,30]. Complementary to this is adsorptive-mediated transcytosis (AMT), which exploits electrostatic interactions between positively charged cationic nanoparticles and the negatively charged endothelial glycocalyx to facilitate entry, although this method often lacks the specificity of ligand-based approaches [31]. More invasive yet highly efficient strategies for bypassing the BBB include focused ultrasound combined with microbubbles, which transiently disrupt tight junctions, as well as the nose-to-brain route, which circumvents the BBB entirely by exploiting the olfactory and trigeminal neural pathways to deliver therapeutics directly to the cerebrospinal fluid [32,33]. Taken together, the heterogeneous integrity of the BBB—particularly at the invasive tumor rim—creates a narrow design window in which nanoparticle size and surface properties become critical determinants of effective drug delivery.

### 2.2. Physicochemical Optimization (Passive Targeting)

Improving the permeation of inorganic nanoparticles involves two parallel approaches: optimizing the particle’s physicochemical properties to favor passive or adsorptive entry, and applying external physical stimuli to transiently disrupt the barrier’s integrity. The geometric and electrostatic properties of an inorganic nanoparticle are the primary determinants of its biodistribution and interaction with the endothelial glycocalyx. In summary, passive targeting alone is insufficient for reliable BBB penetration in glioblastoma, highlighting the need for nanoscale designs that complement EPR-like accumulation with active or physical transport mechanisms.

#### 2.2.1. The “Size Effect”

The literature consensus indicates that smaller nanoparticles generally penetrate the BBB more effectively, though the optimal size range is nuanced. Particles up to 100 nm can theoretically cross a disrupted BBTB via the enhanced permeability and retention (EPR) effect, but penetrating the intact BBB at the tumor margin requires much smaller sizes or active transport [34,35]. The diameter of silica carriers, like mesoporous silica nanoparticles (MSNs), is a critical variable. Comparative studies of PEGylated silica nanoparticles (25, 50, and 100 nm) showed that the 25 nm particles crossed the BBB most efficiently and accumulated in brain capillary endothelial cells. These smaller particles better infiltrated the hypoxic core of glioblastomas, a region often impervious to larger therapeutic vectors due to high interstitial pressure [36]. The 25 nm doxorubicin (DOX) loaded PEGylated MSN (DOX@RMSN25-PEG-TA) achieved a 6-fold increase in brain accumulation compared to free doxorubicin and extended survival in murine models by over 28% [37].

Pushing the size limit further, ultrasmall gold nanoparticles (AuNPs, ~2 nm) have been studied for their ability to cross biological barriers that block larger particles [38,39,40]. These particles are small enough to potentially traverse paracellular spaces or specialized ion channels in the endothelial membrane. In 3D glioblastoma organoid models, which replicate the complex cellular architecture of the tumor, 2 nm AuNPs functionalized with doxorubicin demonstrated deep penetration and high uptake by tumor cells, whereas free doxorubicin failed to penetrate the organoid barrier [38].

Similar to ultrasmall AuNPs, neodymium-doped black phosphorus quantum dots (BPNd) utilize their ultrasmall size to cross the BBB. Their small size allows rapid diffusion and accumulation in the brain, enabling advanced GBM diagnostics such as NIR-II fluorescence imaging [41]. Taken together, these studies indicate that ultrasmall nanoparticles (approximately 2–25 nm) offer a critical advantage for penetrating the intact BBB at the invasive tumor rim, where passive EPR-driven accumulation is insufficient. Particle size, therefore, represents a primary design parameter that directly governs access to infiltrative glioma cells.

#### 2.2.2. Surface Topology: The Role of Roughness

Beyond simple dimensions, the texture of the nanoparticle surface has emerged as a novel lever for permeability modulation. Biological entities like viruses can also utilize surface spikes to engage cell receptors and mechanical entry.

A pivotal study highlighted the impact of nanoscale roughness. Researchers synthesized “virus-like” silica nanoparticles (VSNP) with a rough, spiky surface and compared them to smooth Stöber silica nanoparticles of the same hydrodynamic diameter (~60 nm). The rough VSNPs exhibited a dramatically stronger interaction with endothelial cells. At a concentration of 1 mg/mL, the VSNPs induced a transient opening of endothelial tight junctions, reducing transendothelial electrical resistance by 2.7-fold and increasing the permeability of the barrier to co-administered macromolecules by 1.9-fold [42]. Furthermore, there are studies suggesting that shorter spike lengths cause more rapid BBB penetration than the longer ones [43]. Collectively, these findings suggest that nanoscale surface roughness enhances endothelial interactions and transient barrier permeability, providing an additional, geometry-driven mechanism to improve BBB penetration beyond size alone.

#### 2.2.3. Surface Charge: Zeta Potential

The electrical charge of the nanoparticle dictates its initial adsorption to the endothelial surface. The luminal surface of cerebral capillaries is lined with a negatively charged glycocalyx, which means that positively charged (cationic) nanoparticles can leverage AMT via electrostatic attraction [44].

Cationic surface engineering is applied across various inorganic types. For instance, chitosan-coated selenium nanoparticles (Cs-SeNPs) were engineered to carry a highly positive zeta potential (approximately +47 mV). This charge facilitated robust interaction with the BBB endothelium, allowing the particles to cross and subsequently inhibit glioma cell migration and invasion by downregulating MMP-2 and MMP-9 [45]. Furthermore, chitosan-coated superparamagnetic iron oxide nanoparticles (CS-DX-SPIONs) with a positive zeta potential of +19.2 mV showed significantly higher uptake by glioma cells compared to neutral or negatively charged counterparts [46]. Overall, a positively charged surface promotes BBB interaction via adsorptive-mediated transcytosis; however, excessive cationic charge may compromise biocompatibility, highlighting the need for careful optimization between transport efficiency and cytotoxicity.

#### 2.2.4. Surface Chemistry (Stealth and Solubility)

Surface engineering with polyethylene glycol (PEG) or biomimetic cell membrane coatings is currently considered the gold standard for reducing immunogenicity and prolonging the systemic circulation of inorganic nanocarriers [47,48]. The process of PEGylation establishes a steric barrier that limits this protein adhesion. Without such modification, nanoparticles are susceptible to the nonspecific adsorption of plasma proteins, known as protein corona formation, which triggers macrophage uptake and rapid clearance, as proven on carbon nanotubes (CNTs) among others [49]. By inhibiting the formation of the protein corona, PEGylation effectively increases the likelihood of nanoparticles reaching the tumor [47,50]. In summary, stealth surface modifications such as PEGylation are essential for prolonging systemic circulation and minimizing premature clearance, thereby increasing the probability of effective tumor delivery despite not directly enhancing BBB translocation.

Taken together, these physicochemical parameters—size, topology, surface charge, and chemistry—must be co-optimized, as no single feature alone is sufficient to ensure reliable BBB penetration in GBM.

As stated above, the most effective particle is one that integrates an ultrasmall inorganic core for deep penetration with a stimuli-responsive shell. This combination allows the particle to act as an agent that remains stable during circulation but becomes aggressively cytotoxic only upon entering the glioblastoma’s unique biochemical landscape (Table 1).

### 2.3. Ligand Functionalization (Active Targeting)

While physical disruption provides a general entry route, surface modification with high-affinity ligands enables active targeting of both the BBB and the tumor [51]. This strategy leverages the receptor-mediated transcytosis (RMT) mechanism for active targeting [52,53]. Peptides are preferred for inorganic nanoparticle functionalization due to their small size, high stability, and ease of conjugation, often using thiol-maleimide chemistry [54].

#### 2.3.1. Angiopep-2

Angiopep-2 is a peptide derived from the Kunitz domain of human aprotinin that targets the low-density lipoprotein receptor-related protein 1 (LRP1), highly expressed on both BBB endothelium and glioblastoma cells, providing a dual-targeting mechanism [55].

In a landmark study, ultra-small iron oxide nanoparticles (~3.3 nm core) were conjugated with angiopep-2, forming Fe_3_O_4_-ANG nanoparticles [56]. These nanoparticles showed excellent colloidal stability and high T_1_-weighted MRI contrast (r_1_ relaxivity = 7.45 mM^−1^ s^−1^). They successfully crossed the BBB and accumulated in orthotopic glioblastoma models, providing clear tumor delineation on MRI and demonstrating the potential for precise GBM diagnosis [56]. Angiopep-2 has also been used to functionalize upconversion nanoparticles (UCNPs), allowing them to cross both the BBB and the blood–CSF barrier, enabling dual-mode MRI and near-infrared (NIR) imaging of intracranial tumors [57]. Among the ligands discussed, angiopep-2 is currently the most clinically advanced, with multiple angiopep-2-conjugated nanotherapeutics having progressed to early-phase clinical evaluation, underscoring its strong translational potential.

#### 2.3.2. Arginine-Glycine-Aspartic Acid

Arginine-Glycine-Aspartic acid (RGD) motif binds specifically to αVβ3 integrins, which are markers of angiogenesis [58], expressed at high levels on the proliferating endothelium of brain tumors and on the glioma cells themselves [59].

Cerium oxide nanoparticles (CeNPs) possess unique redox-cycling properties, which manifest in switching between Ce^3+^ and Ce^4+^, making them potent antioxidants [60]. When functionalized with cyclic RGDfK peptides and loaded with doxorubicin (CeNP + Dox + RGD), these particles effectively crossed the BBB in glioma-bearing mice. More importantly, the RGD-targeting facilitated not just chemotherapy delivery but also immunomodulation. One study showed that such treatment repolarizes tumor-associated macrophages (TAMs) from the pro-tumorigenic M2 phenotype to the anti-tumor M1 phenotype, resulting in a 3-fold increase in survival [61].

On the other hand, USPIOs functionalized with RGD peptides (Fe_3_O_4_-PEG-RGD) were engineered for targeted MRI. In vitro study showed that with a core size of ~2.7 nm, these particles displayed high specificity for glioma cells and a high r1 relaxivity of 1.4 mM^−1^ s^−1^, enabling the sensitive detection of tumors via T1-weighted MRI [62]. RGD-based targeting remains predominantly at a preclinical stage, with translational progress limited by integrin heterogeneity and the risk of off-target vascular interactions.

#### 2.3.3. Chlorotoxin

Chlorotoxin (CTX) is a 36-amino acid peptide derived from the venom of the deathstalker scorpion (*Leiurus quinquestriatus*), which has shown remarkable specificity for glioma cells [63,64]. It targets chloride channels [65,66,67], matrix metalloproteinase-2 (MMP-2) [68], and annexin A2 [69], where the last two are absent in normal brain tissue [68,70].

When it comes to NPs, CTX-conjugated iron oxide NPs loaded with gemcitabine demonstrated enhanced BBB translocation and improved killing of GBM cells compared to the free drug. The CTX modification allowed for tumor-specific accumulation while maintaining the superparamagnetic properties required for MRI monitoring [71]. Also, an in vitro study utilizing chlorotoxin-functionalized MSNs loaded with paclitaxel highlighted a nuanced trade-off. While the CTX functionalization increased the specific toxicity of the drug-loaded particles against U87 cells (likely by directing them to more sensitive intracellular compartments), it actually decreased the total cellular uptake compared to non-targeted particles [72], which suggests that while active targeting increases specificity, ligand density and receptor saturation kinetics must be carefully optimized to maximize payload delivery. Further research, this time with polyethylenimine-entrapped gold nanoparticles (AuPENPs) modified with CTX and labeled with Iodine-131, was conducted. These particles were developed for SPECT/CT imaging, successfully crossing the BBB and targeting specifically glioma cells in a rat model; they validated CTX as a robust ligand for inorganic theranostics [73]. Chlorotoxin exhibits high tumor specificity and has demonstrated clinical feasibility primarily in imaging applications, although its use in nanoparticle-mediated drug delivery remains largely preclinical.

#### 2.3.4. Cell-Penetrating Peptides

Cell-penetrating peptides (CPPs) represent a vast and structurally heterogeneous category of molecules [74,75,76], originally identified within naturally occurring proteins such as the transactivating transcription factor (Tat) of HIV [77]. Over the past decade, these peptides have evolved from biological curiosities into promising vehicles for CNS drug delivery, with their potential currently being investigated in clinical trials such as ESCAPE-NA1 (NCT02930018) [78]. The main mechanism of entry relies on electrostatic interactions: most brain-penetrating CPPs carry an intrinsic positive charge [75] that enables them to engage the negatively charged glycoproteins and proteoglycans of the endothelial glycocalyx [79].

Once bound to the luminal surface, these cationic agents typically traverse the BBB via adsorptive-mediated transcytosis, navigating through anionic microdomains, or by interacting with specific surface proteins to trigger endocytic transport. Although preclinical studies have demonstrated numerous examples of peptide–drug conjugates and fusion constructs [75,76,80], translation to clinical use remains challenging. Brain delivery depends critically on the size and physicochemical properties of the payload, necessitating a tailored, case-by-case evaluation for each therapeutic candidate rather than a universal “one-size-fits-all” approach [81]. Despite their robust cellular uptake, cell-penetrating peptides face significant translational barriers due to limited specificity and concerns regarding systemic distribution.

### 2.4. Physical Modulation of Blood–Brain Barrier

Focused ultrasound (FUS) combined with microbubbles is the most clinically advanced physical method for non-invasive BBB disruption [82,83]. This technique allows delivery of inorganic nanoparticles that would otherwise be blocked by the BBB, regardless of their surface chemistry.

The procedure involves systemically injecting lipid-shelled gas microbubbles (typically 1–10 µm in diameter), which then pass through the acoustic field of a focused ultrasound transducer aimed at the brain tumor and undergo stable cavitation—rhythmic expansion and contraction [84]. This oscillation exerts mechanical shear stress and microstreaming forces on the adjacent endothelial cells. The biological response is transient disassembly of tight junction protein complexes (claudin-5, occludin, ZO-1), creating paracellular pores and stimulating active transcellular vesicle transport [85,86].

A major barrier to FUS adoption is the risk of inertial cavitation (bubble collapse), which can cause vascular damage and hemorrhage [87]. However, recent optimization has shown that low-frequency ultrasound is a safer modality. One study found that 850 kHz ultrasound, coupled with a peak negative pressure (PNP) of 125 kPa, induces safe, reversible BBB openings sufficient to deliver nanoparticles sized 70–100 nm [88]. Moreover, in syngeneic GBM mouse models, this protocol increased accumulation of fluorescently labeled nanoparticles in the tumor 6.7-fold compared to non-FUS controls [88].

Radiosensitization therapy is particularly potent for inorganic radiosensitizers. For example, FUS has been used to enhance the brain uptake of Boron-10 agents for boron neutron capture therapy [89]. Boron-containing nanoparticles used as BNCT agents exploit neutron capture-induced cytotoxicity to generate highly localized radiation damage at the cellular level. As summarized by Li et al., BNCT has progressed into early-phase clinical trials, particularly for gliomas and head-and-neck malignancies, demonstrating encouraging therapeutic responses. However, widespread clinical translation remains limited by the requirement for specialized neutron sources, complex treatment infrastructure, and challenges associated with achieving homogeneous intratumoral boron accumulation, positioning nanoparticle-based BNCT at the interface of advanced preclinical development and early clinical evaluation [90,91].

### 2.5. Drug Release and Intracellular Delivery

While nanoparticle design governs BBB penetration and tumor accumulation, therapeutic efficacy in GBM ultimately depends on efficient intracellular drug release and trafficking.

#### 2.5.1. Controlled and Stimuli-Responsive Release

To minimize systemic toxicity and maximize local efficacy, inorganic nanocarriers are often designed as “zero-premature-release” systems that retain their cargo until triggered by specific stimuli [92]. MSNs, for instance, can be capped with molecular “gatekeepers” that block the pores and prevent drug leakage during circulation [93]. These gates open only in response to the unique biochemical signature of the GBM microenvironment, such as acidic pH caused by the Warburg effect or elevated intracellular glutathione (GSH) levels, which can cleave the disulfide bonds holding the caps in place [93]. Beyond internal triggers, external stimuli provide clinician-controlled precision. Iron oxide nanoparticles (IONPs) generate localized heat under alternating magnetic fields, melting thermosensitive lipid or polymer coatings to release the drug at the desired time [94].

#### 2.5.2. Endocytosis and Cellular Uptake

Once at the tumor site, nanoparticles must cross the glioma cell membrane, a process strongly influenced by their physicochemical surface properties. While passive accumulation via the Enhanced Permeability and Retention (EPR) effect allows for some uptake, active targeting strategies that functionalize particle surfaces with ligands—such as transferrin, RGD peptides, or antibodies—significantly enhance internalization via receptor-mediated endocytosis [95,96]. Surface charge is also critical: cationic (positively charged) particles usually enter cells faster due to electrostatic attraction to the negatively charged membrane, but their charge must be carefully balanced to avoid cytotoxicity, often requiring charge-reversal strategies that activate only in the acidic tumor environment [97].

#### 2.5.3. Intracellular Trafficking and Endosomal Escape

**Figure 2:**
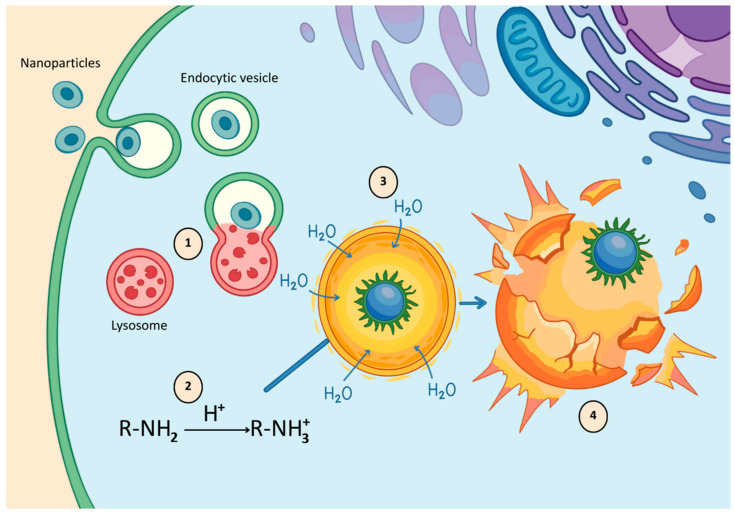
Endosomal escape via the proton sponge effect: acidification induces ionization of functional groups, leading to osmotic swelling, endosomal rupture, and cytosolic release of nanoparticles.

After internalization, the main barrier to therapeutic efficacy is entrapment within endosomes, which mature into acidic, enzyme-rich lysosomes that can degrade sensitive payloads such as siRNA or proteins [98]. To avoid this “lysosomal death,” inorganic nanoparticles employ unique escape mechanisms (Figure 2). Materials such as calcium phosphate (CaP) and manganese dioxide (MnO_2_) are pH-sensitive. They dissolve rapidly in the acidic late endosome, raising ionic concentration and causing vesicle rupture via osmotic pressure (the “proton sponge” or osmotic lysis effect; Figure 2) [99].

Alternatively, photothermal agents like AuNPs can be irradiated to physically disrupt the endosomal membrane through localized heating or vapor bubble formation, ensuring the cargo is delivered intact into the cytosol [100].

#### 2.5.4. Intracellular Delivery and Therapeutic Outcome

Efficient intracellular delivery is a key determinant of therapeutic efficacy in GBM nanomedicine. After cellular uptake, many nanoparticles remain trapped within endolysosomal compartments, which limits drug bioavailability and reduces therapeutic impact. Systems that enable controlled intracellular release and efficient endosomal escape consistently show higher cytotoxicity than formulations lacking these features. pH- and redox-responsive nanocarriers exploit the acidic and reductive intracellular environment of glioma cells to trigger payload release at the desired site. When combined with endosomal escape mechanisms, these systems significantly increase tumor cell apoptosis and enhance intracellular drug availability. Importantly, multiple preclinical studies report prolonged median survival in murine GBM models treated with such responsive nanocarriers compared with free drugs or non-responsive formulations [101,102,103,104]. These findings demonstrate that tumor accumulation alone is insufficient. Instead, therapeutic outcomes depend on the ability of nanoparticles to overcome intracellular barriers and deliver bioactive cargo to its site of action. Accordingly, the rational design of inorganic nanocarriers for GBM must integrate intracellular release and escape mechanisms as core determinants of therapeutic success.

## 3. Inorganic Nanoparticles Used in GBM Therapy

Nanotechnology resides at the nexus of physics, chemistry, and biology, governing materials defined by a dimension between 1 and 100 nanometers. But this is not merely a matter of scale; it represents a shift in material behavior. Unlike their bulk counterparts, NPs boast an immense surface-to-volume ratio. This geometric quirk unlocks heightened chemical reactivity and unique quantum optical behaviors [101]. This is called the “nanoscale advantage.” It permits precise engineering of surface topology, allowing the conjugation of specific ligands, peptides, antibodies, and aptamers, among others, which target pathological tissues while sparing healthy ones [102].

When addressing CNS disorders, the cardinal virtue of these particulates is their size. Being significantly smaller than the tight junctions of the BBB, appropriately designed NPs (typically <100 nm) [34] can traverse this neurovascular fortress. Acting as molecular “Trojan horses,” they deliver therapeutic payloads directly into the TME [103,104]. Moreover, by evading clearance by the reticuloendothelial system (RES), they achieve prolonged systemic circulation, creating a therapeutic window that standard pharmacotherapy cannot match [105].

Broadly, the nanomedicine landscape bifurcates into organic and inorganic families. Organic platforms, such as liposomes, dendrimers, and polymeric micelles, have long dominated the field, valued for their biodegradability and ability to protect fragile biomolecules such as mRNA [106]. While both categories contribute significantly to GBM management, this review will focus exclusively on inorganic nanoparticles. This focus is justified by the intrinsic physical properties of inorganic materials, which extend beyond mere drug delivery. Unlike organic nanoparticles, inorganic ones often possess inherent magnetic, plasmonic, or radiological properties that facilitate theranostics—the convergence of diagnosis and therapy [107]. AuNPs leverage surface plasmon resonance to act as radiosensitizers [94]. Coupled with superior structural rigidity that supports complex functionalization [108], they represent a distinct class of tools. Although safety concerns regarding accumulation and oxidative stress require careful evaluation [109], the ability of inorganic NPs to respond to external stimuli (light, magnetism, radiation) positions them at the forefront of multimodal glioma therapy.

### 3.1. Carbon-Based Platforms

#### 3.1.1. Quantum and Carbon Dots

**Figure 3:**
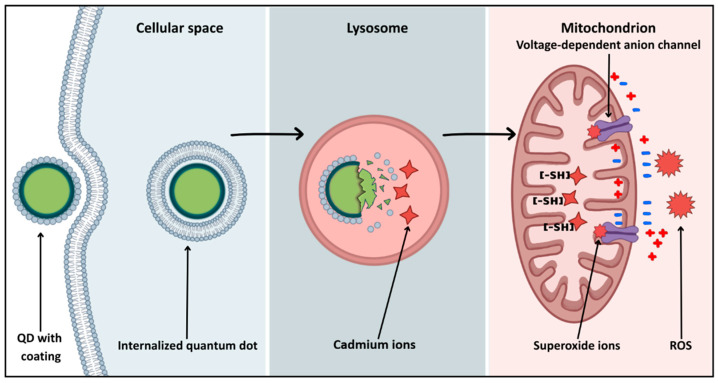
Intracellular pathway of QD-induced mitochondrial dysfunction.

In nanomedicine, ‘quantum dots’ include two classes of materials with similar optical properties but vastly different safety profiles. Classic quantum dots (QDs) are semiconductor nanocrystals, for example CdSe and CdTe, which are prized for their exceptional optical properties. However, it must not be forgotten that their brilliance comes at a biological cost hidden in a core composed of heavy metals. Upon cellular internalization, the acidic environment of endosomes and lysosomes can degrade the particle’s surface coating [110], triggering the release of free cadmium ions (Figure 3). These ions, on the other hand, show high affinity for mitochondrial protein sulfhydryl groups, precipitating the collapse of mitochondrial membrane potential [111]. Such interaction leads to respiratory chain blockage and rapid reactive oxygen species (ROS) generation, inducing apoptosis of neurons and glial cells [111] (Figure 3).

In stark contrast stand Carbon Dots (CDs) and Graphene Quantum Dots (GQDs). Their intrinsic biocompatibility stems from a chemically inert carbon core rather than potentially toxic metals [112], like in the case of QDs. Their safety is further reinforced by favorable pharmacokinetics as CDs with a hydrodynamic diameter smaller than 5.5 nm effectively utilize the renal clearance pathway (discussed in Section 4), minimizing long-term CNS retention [113].

The therapeutic utility of CDs in GBM is inextricably linked to chemical modification, or “doping”. Pure CDs typically emit light in the blue or green spectrum, which corresponds to wavelengths that are easily absorbed by tissue and therefore are suboptimal for deep brain imaging [114]. To overcome this, nitrogen or sulfur atoms are introduced into the carbon crystal lattice. Nitrogen possesses a lone pair of electrons that adds energy levels between the valence and conduction bands. This effectively red-shifts the particle’s absorption and emission into the near-infrared (NIR) spectrum, enabling imaging in the second biological window (NIR-II, 1000–1700 nm) [115,116].

The advantage of NIR-II over the traditional NIR-I windows is grounded in the fundamental laws of tissue optics, Rayleigh scattering theory, in that case, which states that photon scattering by tissue particles is inversely proportional to the fourth power of the wavelength [117]. Consequently, shifting detection from 800 nm (NIR-I) to 1300 nm (NIR-II) drastically mitigates scattering by brain lipids and skull bone [118], allowing for precise tumor localization through the skull [119,120].

CDs and QDs engineered for NIR-II emission allow for achieving penetration depths of a centimeter while maintaining a high signal-to-noise ratio [121]. This characteristic enabled non-invasive monitoring of tumor growth through the intact skull of mice in orthotopic models [121], promising a chance of detection of micro-scale tumor infiltrations hidden under a layer of healthy tissue during resection in clinical conditions.

This optical engineering addresses a critical challenge in neuro-oncology: the precise delineation of tumor margins. While standard fluorophores like 5-aminolevulinic acid (5-ALA) or indocyanine green (ICG) are clinically useful, they suffer from significant physicochemical limitations, primarily susceptibility to photobleaching and light emission in the visible (400–700 nm) or NIR-I range [122]. Carbon nanodots feature a rigid core structure that provides outstanding photostability compared to organic dyes, which degrade rapidly under strong operating room lights [123]. This stability ensures a consistent fluorescent signal throughout long neurosurgical procedures [124]. Such stability gives surgeons an advantage of “illuminating” resection margins until the very last minute of surgery, minimizing the risk of leaving minimal residual disease, which is one of the primary drivers of GBM recurrence [125].

Beyond imaging, doping also fundamentally alters the mechanism of ROS generation. In the classical photodynamic therapy (PDT) model (type II), an excited photosensitizer transfers energy to triplet oxygen to create singlet oxygen. However, this mechanism often fails in the hypoxic core of GBM tumors where oxygen is scarce. Modern sulfur and nitrogen-doped CDs are designed to circumvent this limitation by promoting a type I mechanism. Instead of relying on energy transfer, they transfer an electron directly to biological substrates or oxygen, generating superoxide radicals. These radicals remain lethal even in low-oxygen environments, rendering doped CDs a powerful weapon against the otherwise recalcitrant, hypoxic glioma core [126,127,128]. Compared to classical cadmium-based quantum dots, carbon dots and nanodiamonds offer a markedly improved safety profile, albeit at the cost of reduced optical brightness or limited BBB penetration, respectively.

#### 3.1.2. Carbon Nanotubes

CNTs are defined by their unique cylindrical geometry and high aspect ratio and are broadly categorized into single-walled (SWCNTs) and multi-walled (MWCNTs) varieties [129]. While they inherit the exceptional mechanical and thermal properties of graphite [130], their application in GBM faces two major hurdles, which are toxicity and insolubility. The hydrophobic carbon core tends to agglomerate in aqueous environments, posing a risk of length-dependent toxicity reminiscent of asbestos fibers [131]. Consequently, the translation of CNTs into clinical practice hinges entirely on solving these dispersion and biocompatibility issues through rigorous surface functionalization [132].

Safety is nuanced because shorter MWCNTs generally exhibit a better profile than their single-walled counterparts, creating a preference for their use in in vivo applications [133,134]. To survive systemic circulation, CNTs must be hydrophilized, often via covalent modification with carboxylic acid (-COOH) groups or non-covalent coating with polymers such as PEG. This engineering is essential not only to maintain stable dispersion in plasma and cerebrospinal fluid but also to bypass rapid clearance by RES.

Once stabilized, the massive specific surface area of CNTs allows for substantial drug loading capacity (DLC) and dense targeting functionalization [135]. For aromatic hydrophobic drugs like Doxorubicin, Paclitaxel, or Camptothecin, the primary loading mechanism is non-covalent “stacking” between the drug’s rings and the CNT walls, achieving very high DLC [136,137,138]. For less aromatic agents like TMZ, covalent loading is typically employed.

Crucially, these payloads are not just carried but intelligently released. Systems exploit the acidic nature of glioma lysosomes to hydrolyze acid-labile bonds [139] or utilize high intracellular GSH levels to sever disulfide linkages holding the cargo. Furthermore, functionalization with cationic polymer NPs, such as PEI, enables the “proton sponge effect.” This phenomenon involves buffering the acidic environment of the endosome, causing organelle swelling and rupture, which releases genetic payloads such as siRNA directly into the cytoplasm before lysosomal degradation occurs [140].

Beyond drug delivery, CNTs act as potent physical agents in photothermal therapy (PTT). They are exceptionally efficient at converting NIR light (700–1000 nm) into heat via non-radiative exciton decay [141]. Besides thermally ablating cancer cells, this process also drives a powerful synergistic effect. The heat generated during PTT accelerates the release of loaded chemotherapeutics, temporarily increases vascular permeability, and sensitizes cells to the chemotherapy itself [142,143]. Moreover, nanotubes engineered with crystal lattice defects, such as nitrogen-vacancy centers, offer stable fluorescence for long-term tracking and, together with their quantum properties, act as nanosensors for intracellular thermometry, allowing precise, real-time optimization of PTT protocols [144,145].

Emerging research suggests CNTs may remodel immunosuppressive TME. Appropriate functionalization can repolarize TAMs from a pro-tumorigenic M2 state to a pro-inflammatory and cytotoxic M1 state, representing a novel therapeutic strategy [146].

#### 3.1.3. Nanodiamonds

In the crowded landscape of engineered nanomaterials for brain tumor therapy, nanodiamonds (NDs) stand out as a uniquely promising platform. Physically, these are tiny carbon NPs, typically 4–5 nm in size, constructed from a chemically inert diamond (sp^3^) core wrapped in a shell of graphitic or amorphous carbon (sp^2^) [147]. It is this dual structure that unlocks their potential: the outer shell enables rich surface chemistry and functionalization [147], while the core provides stability. The modifiable surface allows for the conjugation or adsorption of a wide variety of therapeutic molecules, such as drugs, nucleic acids, and targeting ligands, which is crucial for creating multifunctional carriers that remain stable under physiological conditions [148]. Unlike metal-based NPs, the chemically inert carbon core of NDs offers a significant safety advantage, as it does not release metal ions and therefore poses no risk of leaking potentially neurotoxic species that could cause chronic toxicity [149].

The therapeutic potential of this platform has already been tested in preclinical glioma models. For instance, when NDs were conjugated with doxorubicin (ND–DOX) and administered using convection-enhanced delivery (CED), enhanced uptake, prolonged tumor retention, increased tumor cell death, and reduced toxicity to healthy brain tissue were observed compared with free doxorubicin [147,150]. Beyond therapy alone, the capacity to load imaging agents alongside drugs positions NDs as powerful tools for combined therapeutic and diagnostic strategies (theranostics), potentially allowing clinicians to treat tumors while monitoring delivery in real time [148].

Initial safety data on NDs are encouraging, as both in vitro and in vivo studies suggest a favorable biocompatibility profile characterized by low cytotoxicity, minimal hemolytic activity, and limited induction of inflammatory responses [149]. Nevertheless, significant gaps remain in the understanding of the long-term fate of these particles, particularly with respect to biodistribution, biodegradation mechanisms, and potential accumulation in off-target organs after repeated administration [148]. Additionally, while local methods such as CED have proven effective, crossing the BBB following systemic administration remains far more challenging; nevertheless, strategies such as receptor-targeted functionalization are being explored to address this limitation [151].

### 3.2. Silica Nanoparticles

Among inorganic nanocarriers, MSNs have emerged as one of the most versatile and well-studied tools in nanomedicine [152]. Valued for their chemical stability and the precision with which they can be synthesized, MSNs are particularly promising for tackling GBM [153,154]. By offering a platform that integrates precise drug delivery with diagnostic capabilities, MSNs open the door to advanced theranostic strategies [155,156]. Typically produced via the Stöber method or using surfactant-based templates, these particles can be engineered with remarkable uniformity and strictly defined porosity [157,158]. It is this unique architecture—specifically their massive surface area and tunable pore sizes—that makes them so effective [50]. They can easily sequester both hydrophobic and hydrophilic compounds [159]. Moreover, the ability to functionalize the internal pore surfaces separately from the outer shell provides chemists with a highly adaptable canvas for therapeutic design [50,158].

Delivery of these carriers to the tumor relies largely on the EPR effect, although surface ligands can further enhance targeting. Indeed, in animal models of GBM, multicomponent MSNs have demonstrated efficient accumulation within tumor tissue [155]. However, success is often governed by geometry: precise tuning of particle size, shape, and surface properties is critical for optimizing distribution and retention within the malignancy [157].

Moving beyond simple carriers, MSNs are increasingly being deployed as complex, multifunctional systems [160]. For example, wrapping an iron oxide core in a mesoporous silica shell (Fe@MSN) yields particles that deliver drugs while enabling real-time biodistribution tracking via MRI—a strategy shown to inhibit tumor growth and extend survival in preclinical studies [155]. Recent innovations have combined chemotherapy with immunotherapy. In one approach, immuno-MSNs loaded with STING agonists were taken up by antigen-presenting cells in the TME, boosting immune infiltration and delaying tumor growth in vivo [161].

Finally, these platforms can be engineered to be “smart,” releasing their cargo only in response to specific biological triggers. Some formulations utilize the acidic environment of lysosomes to trigger the pH-responsive release of cytotoxins like paclitaxel [72], while others use redox-responsive systems to exploit the elevated GSH levels found in tumor cells to cleave disulfide bonds and free the drug [162]. MSNs have also proven effective in PDT [163]. For instance, particles loaded with hydrophilic photosensitizers such as chlorin e6 generate significant ROS upon irradiation, driving cell death in aggressive glioma lines [164].

### 3.3. Metallic and Metal Oxide Platforms

#### 3.3.1. Metal Oxide Nanoparticles

Within the oncological arsenal against GBM, metal oxide nanoparticles (MONPs) represent a distinct and versatile class of materials. Unlike organic NPs, MONPs are defined by their intrinsic physicochemical properties, ranging from magnetic susceptibility to catalytic activity, which unlock multimodal therapeutic strategies [47,165].

At the vanguard of this neuro-oncological frontier stand superparamagnetic iron oxide nanoparticles (SPIONs), primarily composed of magnetite (Fe_3_O_4_) and maghemite (γ-Fe_2_O_3_) [166]. SPIONs are particularly valuable because of their specific magnetic domain structure, which results in the exhibition of magnetic properties only in the presence of an external field, thereby preventing agglomeration in the bloodstream—a critical safety feature that reduces the risk of embolization [47,165]. When appropriately engineered with targeting ligands like peptides or antibodies, SPIONs can traverse the BBB in experimental models, gathering within the tumor tissue while sparing the healthy brain parenchyma [46,167].

The clinical utility of SPIONs is defined by their dual role as theranostic agents. Therapeutically, their most advanced application is interstitial magnetic hyperthermia. After exposure to an alternating magnetic field, SPIONs dissipate heat through Néel and Brownian relaxation, which raises the local temperature to 42–46 °C and induces irreversible protein denaturation and heat shock protein expression within neoplastic tissue [47,168].

Diagnostically, SPIONs act as potent T_2_-weighted contrast agents. Their high magnetic susceptibility creates local field inhomogeneities that shorten relaxation times, producing hypointense (dark) MRI signals and enabling real-time monitoring of biodistribution and therapeutic response, thereby fulfilling the need for image-guided therapy [165,169].

Beyond the magnetic allure of iron-based systems, the research lens is widening to encompass other metal oxides that wield intrinsic cytotoxic or redox-modulating powers. Zinc oxide nanoparticles (ZnO-NPs), for instance, are well regarded for their ability to generate ROS, driving a state of profound oxidative stress [170]. What makes them particularly effective is their intracellular instability, which manifests as partial dissolution of ZnO-NPs and the release of a sudden flux of Zn^2+^ ions. This influx disrupts cellular homeostasis, forcing the cell into a caspase-activated apoptotic pathway and leading to membrane potential collapse [171]. This lethality is well-supported by experimental evidence in glioma cell lines like U87MG, where ZnO-NPs have been shown to trigger significant DNA fragmentation and a sharp drop in cell viability, positioning them as promising agents for both direct cytotoxicity and drug delivery [172,173].

Nevertheless, perhaps the most biologically fascinating candidate is cerium oxide (CeO_2_), or nanoceria. It operates as a molecular chameleon, with its function dictated entirely by pH. In the acidic TME, CeO_2_ acts as a pro-oxidant that amplifies oxidative stress and destroys cancer cells; in contrast, at the near-neutral pH of healthy brain tissue, it undergoes a functional reversal, mimicking protective enzymes such as superoxide dismutase and scavenging free radicals, thereby shielding neurons from radiation-induced collateral damage [174,175].

Despite this immense potential, the translation of MONPs into clinical practice faces significant challenges, particularly with respect to clearance and neurotoxicity. Non-biodegradable particles, such as certain forms of TiO_2_ or noble metal-doped oxides, risk accumulating in the brain parenchyma or the RES, potentially triggering chronic inflammation.

#### 3.3.2. Gold Nanoparticles

Recently, AuNPs have emerged as a promising therapeutic platform for the treatment of GBM. Scientific interest in them stems from their versatile physicochemical properties, which allow for surface modification, precise control of size and shape, and exploitation of plasmonic resonance in light-based therapies [38,176]. While traversing the BBB remains a critical hurdle in GBM therapy, surface engineering, such as PEGylation or the attachment of targeting ligands and cell-penetrating peptides, has successfully facilitated BBB transport. This targeted approach enhances accumulation within the TME, effectively delivering chemotherapeutic agents directly to the lesion [177,178].

In the context of radiotherapy, AuNPs function as radiosensitizers, increasing local radiation absorption and stimulating the generation of ROS, thereby amplifying the effects of radiotherapy and resulting in improved tumor growth control [179]. In both in vitro and in vivo models, the combination of AuNPs and radiotherapy demonstrated the ability to induce immunogenic cell death in cancer cells, leading to the release of DAMP molecules and activation of an anti-tumor immune response [180]. Furthermore, some studies link Au@DTDTPA(Gd) with reduced cancer cell invasiveness and migration, suggesting a potential role in mitigating recurrence risks [181]. Nevertheless, the effectiveness of radiosensitization strongly depends on AuNP design, including size, coating, and functionalization. Consequently, only well-optimized configurations (e.g., very small or peptide-targeted AuNPs) lead to significant ROS production and DNA damage [179,182].

Besides radiotherapy, AuNPs are steadily becoming of key importance in PTT, where they convert absorbed light into heat to selectively ablate cancer cells [183]. Gold nanorods (GNRs), owing to their localized surface plasmon resonance, are particularly efficient at this conversion [184]. After laser irradiation, GNRs can induce apoptosis in cancer cells, promoting a milder inflammatory response and potentially supporting the immune response [185]. In animal models, the use of GNRs resulted in tumor mass reduction and prolonged survival [183]. Therapeutic efficacy is further enhanced when AuNPs are paired with photosensitizers for simultaneous photothermal therapy and PDT, or combined with drug carriers to exert synergistic anti-tumor effects [183,186]. AuNPs also hold significant promise as theranostic carriers, enabling both treatment and tumor imaging. In murine models, AuNPs improved CT contrast, with a high tumor-to-normal brain gold ratio (~19:1), which expedited the process of tumor localization and therapy planning [187]. Moreover, they demonstrated high affinity to tumor microvasculature, accumulating there and improving visualization of tumor margins [188].

#### 3.3.3. Silver Nanoparticles

Silver nanoparticles (AgNPs) represent a fascinating shift in the therapeutic landscape by acting not merely as passive carriers but as a multifunctional weapon against GBM with intrinsic lethal capabilities [189,190,191,192,193]. Their primary mode of attack is biochemical warfare at the cellular level. Upon internalization, AgNPs unleash a storm of oxidative stress by generating high levels of ROS. This oxidative burst overwhelms the cancer cell’s defenses, causing mitochondrial dysfunction, a collapse of membrane potential, and ultimately, the triggering of caspase-dependent apoptotic cascades [194].

However, the assault is multipronged. Beyond simple toxicity, AgNPs actively disrupt the reproductive machinery of the tumor by enforcing cell-cycle arrest at the G2/M phase through interference with critical regulatory proteins such as cyclin B1 and Cdc2 [189]. Evidence for this appears in several studies analyzing both in vitro and in vivo models, in which glioma cells stop proliferating, tumors struggle to grow, and tissue analysis reveals a distinct shift toward cell death marked by DNA fragmentation, a spike in pro-apoptotic markers (Bax, p53), and a reduction in survival proteins such as Bcl-2 [189,191,192].

Crucially, AgNPs also function as a force multiplier for existing therapies. They appear to physically destabilize the cellular membrane, increasing permeability and allowing cytotoxic drugs to enter the cell more readily, a mechanism linked to altered lipid structures and enhanced endocytosis [189]. This synergy is potent: 26 nm AgNPs, for instance, have been shown to sensitize U251 cells to TMZ, effectively overcoming chemoresistance [189]. They also act similarly as radiosensitizers, prolonging survival and intensifying DNA damage even in the hypoxic, radio-resistant niches of the tumor [193,194]. Perhaps most surprisingly, this aggression appears to be selective. In U-87MG models, AgNPs suppressed tumor growth by nearly 89% yet showed relatively low toxicity toward healthy astrocytes and fibroblasts [190]. There is even emerging evidence suggesting AgNPs might modulate the inflammatory landscape of the tumor microenvironment, potentially turning the immune system against the glioma [195,196]. However, the path to the clinic is not yet clear, as significant hurdles related to systemic toxicity, long-term biodistribution, and biological stability underscore the urgent need to optimize surface coatings and dosing before these NPs can be safely deployed in patients [197,198]. While silver and zinc oxide nanoparticles exhibit strong intrinsic cytotoxicity, their therapeutic applicability is substantially constrained by systemic toxicity, in contrast to gold and iron oxide nanoparticles, which display more favorable translational profiles. Table 2 summarizes the most important types of inorganic nanoparticles used in GBM research, listing their mechanisms of action, key advantages, potential limitations and toxicity risks, as well as stage of development and overall assessment of therapeutic potential. It provides an overview that allows for comparison of the safety and efficacy of different platforms, from highly cytotoxic nanoparticles to more clinically advanced theranostic systems.

The table summarizes all classes of inorganic nanoparticles discussed in the manuscript, including both the main therapeutic platforms and adjuvant materials used for imaging, targeting, or intracellular delivery. It summarizes the overall therapeutic potential of each nanoparticle class, taking into account efficacy and toxicity considerations. Considering the current balance between therapeutic efficacy, safety, and clinical maturity, gold nanoparticles and iron oxide-based systems appear to be the most promising inorganic platforms for near-term clinical translation, whereas most other nanomaterials remain at an exploratory preclinical stage. Importantly, no single inorganic nanoplatform is universally optimal, and the selection of a given material must be guided by the intended therapeutic mechanism, delivery route, and acceptable safety trade-offs.

## 4. Off-Target Effects

While the engineering of inorganic nanoparticles is often focused on maximizing tumor accumulation, their clinical viability is defined just as critically by their fate in healthy tissues. The persistence of non-biodegradable noble metals and the catalytic potential of metal oxides introduce distinct systemic risks, limiting their use in GBM treatment [199,200,201,202].

Immediately after intravenous administration, nanoparticles (NPs) encounter the reticuloendothelial system (RES), also known as the mononuclear phagocyte system (MPS). This system serves as the primary biological blockade against treatment. Specialized macrophages in the liver (Kupffer cells) and spleen sequester the vast majority of the injected payload, acting as a sink that frequently attenuates the effective dose reaching the brain to approximately 0.7% [196].

The liver functions as the dominant clearance organ, employing a size-dependent sorting mechanism. Kupffer cells rapidly phagocytose particles exceeding 100 nm [197]; conversely, nanoparticles smaller than 100 nm may permeate the hepatic sinusoidal fenestrae (approximately 100–150 nm), entering the space of Disse to interact directly with hepatocytes and Ito cells [203,204]. Such interactions are of vital clinical significance; for instance, up to 73% of administered iron oxide NPs accumulate in the liver shortly after injection [205]. Although iron oxide is generally biocompatible and metabolically integrated into ferritin or hemoglobin, such massive, transient accumulation poses a risk of saturating macrophage capacity or inciting localized oxidative stress [205,206].

Particles that evade hepatic capture must navigate the spleen, which filters the blood for senescent cells and particulates. The splenic architecture requires passage through inter-endothelial slits approximately 200 nm wide; hence, rigid, non-deformable inorganic nanoparticles are frequently trapped within this filtration meshwork [113,207].

To mitigate the risks of chronic inflammation and heavy metal toxicity associated with long-term somatic retention, renal excretion is the preferred elimination pathway. However, this route is guarded by the glomerular filtration barrier, which imposes a strict hydrodynamic size cutoff of approximately 5.5 nm [113], thereby creating a fundamental design conflict. Inorganic nanoparticles larger than 6 nm are generally excluded from urinary excretion, destined instead for hepatobiliary clearance or indefinite retention [208]. While these larger particles, such as 50 nm AuNPs, benefit from superior tumor retention via the EPR effect, their inability to leave the body poses significant safety concerns.

The development of ultrasmall gold nanoparticles (e.g., 3 nm) represents a promising solution to this paradox. As discussed in the context of passive targeting, these agents effectively accumulate in gliomas while simultaneously offering a favorable clearance profile, with approximately 50% of the administered dose excreted within 24 h [209]. Nevertheless, this pharmacokinetic shift relocates the potential toxicity burden from the liver to the kidneys. Here, surface engineering is paramount: while cationic ultrasmall particles may adhere to the negatively charged glomerular basement membrane and induce nephrotoxicity, zwitterionic coatings can facilitate rapid, non-toxic passage through the renal filter [210].

## 5. Conclusions

The therapeutic landscape of GBM is undergoing a major transformation. Inorganic nanotechnology is evolving from simple drug carriers into multifunctional therapeutic platforms. Although the BBB still blocks approximately 98% of small-molecule drugs, nanoparticles with carefully engineered size and surface properties can exploit passive diffusion, active transport, and receptor-mediated pathways. Inorganic nanomaterials offer a distinct “nanoscale advantage.” Their intrinsic magnetic, optical, and catalytic properties enable true theranostic strategies. These platforms allow clinicians to visualize tumor boundaries while simultaneously inducing localized tumor ablation through hyperthermia or radiosensitization. Despite this promise, clinical translation remains challenging. A central issue is the pharmacokinetic paradox: nanoparticles must be large enough to avoid rapid renal clearance, yet small enough to penetrate deep brain parenchyma. Future advances will likely depend on smart surface chemistries that remain inert during circulation but become selectively active within the biochemical environment of glioblastoma. Nevertheless, the number of FDA-approved inorganic nanomedicines for GBM remains extremely limited. This gap persists despite decades of encouraging preclinical data. One major concern is long-term accumulation and toxicity. Unlike biodegradable organic systems, most inorganic nanoparticles are not degradable. They can accumulate in the liver, spleen, or brain, raising the risk of chronic inflammation and delayed neurotoxicity. Scalability and reproducibility present additional barriers. While MSNs can be synthesized with high uniformity, maintaining strict batch-to-batch consistency in porosity or surface chemistry at an industrial scale is technically demanding. Furthermore, inorganic nanoparticles often act as hybrid agents. They function both as drug carriers and responders to physical stimuli such as heat or radiation. This dual role complicates clinical trial design and regulatory safety assessment. Finally, unmodified nanoparticles are prone to nonspecific protein adsorption, leading to protein corona formation. This process promotes macrophage uptake and rapid systemic clearance. Even small variations in surface modification can significantly alter the effective dose delivered to the tumor in human patients.

## Figures and Tables

**Table 1:** Design principles for nanoparticle-mediated BBB penetration.

Feature	Desired Property	Rationale for Effectiveness	Reported BBB Penetration Outcome
Core size	<25 nm	Enables penetration of intact BBB via paracellular transport or specialized endothelial pathways at the invasive tumor rim.	Enhanced accumulation in invasive tumor margins
Surface texture	Nanoscale roughness	Promotes transient disruption of endothelial tight junctions through enhanced mechanical interaction.	Increased BBB permeability in preclinical models
Zeta potential	Positive	Facilitates electrostatic interaction with the negatively charged endothelial glycocalyx, promoting AMT.	Improved endothelial uptake and transcytosis
Surface coating	PEG	Reduces protein corona formation and prolongs systemic circulation time.	Higher brain-to-plasma ratios

**Table 2:** Summary of Inorganic Nanoparticles for GBM: Mechanisms, Advantages, Limitations, Development Stage, and Qualitative Therapeutic Potential.

Type of Nanoparticle	Mechanism of Action	Primary Advantages	Primary Limitations/Toxicity Risks	Development Stage	Overall Therapeutic Potential (Qualitative)
Carbon Dots (CDs)	NIR-II imaging; Type I ROS generation	High biocompatibility; Excellent photostability; Efficient renal clearance	Native blue/green emission of undoped CDs is suboptimal for deep-tissue imaging	Preclinical	Imaging-oriented platform with emerging theranostic potential
Classical Quantum Dots (Cd-based QDs)	Fluorescence imaging	Exceptional brightness and photostability	Release of toxic Cd^2+^ ions; Mitochondrial dysfunction; Neurotoxicity	Preclinical	High imaging performance limited by severe toxicity concerns
Carbon Nanotubes (CNTs)	Drug π–π stacking; Photothermal therapy; Proton sponge effect	Extremely high surface area; Efficient light-to-heat conversion	Hydrophobicity and aggregation; Fiber-like, asbestos-type toxicity risk	Preclinical	High therapeutic efficacy constrained by safety and biodegradability issues
Nanodiamonds (NDs)	Controlled drug adsorption and release	Chemically inert carbon core; No metal ion leakage; Good biocompatibility	Limited BBB penetration following systemic administration	Preclinical	Biocompatible drug carrier with delivery-related limitations
Mesoporous Silica Nanoparticles (MSNs)	Pore sequestration; Stimuli-responsive (“smart”) gating	High structural uniformity; Tunable porosity; High drug-loading capacity	Requires precise size and surface optimization for effective brain retention	Preclinical	Versatile delivery platform with optimization-dependent efficacy
Iron Oxide Nanoparticles (SPIONs)	Magnetic hyperthermia; T_2_-weighted MRI contrast	Magnetic targeting; Real-time imaging and therapy monitoring	Risk of macrophage overload; Local oxidative stress in RES organs	Clinical/Preclinical	Clinically validated imaging platform with adjunctive therapeutic utility
Zinc Oxide Nanoparticles (ZnO-NPs)	ROS burst; Intracellular dissolution releasing Zn^2+^	Strong intrinsic cytotoxicity; No external trigger required	Poor intracellular stability; Excessive oxidative stress	Preclinical	Potent anticancer activity limited by poor controllability
Cerium Oxide Nanoparticles (CeO_2_)	Redox cycling (Ce^3+^/Ce^4+^)	pH-dependent antioxidant (healthy tissue) and pro-oxidant (tumor) behavior	Risk of long-term accumulation in brain parenchyma or RES	Preclinical	Context-dependent therapeutic effects with accumulation concerns
Gold Nanoparticles (AuNPs)	Radiosensitization; CT contrast; Photothermal therapy	Precise size/shape control; Strong radiosensitization; CT enhancement	Potential long-term retention if size exceeds renal clearance threshold (~6 nm)	Clinical/Preclinical	Clinically advanced theranostic platform with size-dependent safety
Silver Nanoparticles (AgNPs)	Mitochondrial dysfunction; G_2_/M cell-cycle arrest	Multi-mechanistic anticancer activity	High systemic toxicity; Requires extensive surface-coating optimization	Preclinical	Highly potent but toxicity-limited therapeutic candidate
Black Phosphorus Quantum Dots (BPQDs)	NIR-II imaging; Photothermal/photodynamic effects	Excellent NIR absorption; Efficient BBB penetration at ultrasmall size	Intrinsic chemical instability; Oxidative degradation	Preclinical	Promising NIR theranostic platform limited by stability issues
Upconversion Nanoparticles (UCNPs)	NIR-to-visible/NIR emission; Dual-mode imaging	Deep-tissue optical imaging; Low autofluorescence	Low quantum efficiency; Complex synthesis	Preclinical	Specialized imaging platform with limited standalone therapeutic value
Calcium Phosphate/MnO_2_ Nanoparticles	Endosomal escape via proton-sponge or osmotic rupture	Efficient cytosolic delivery; pH-responsive degradation	Limited in vivo stability; Restricted standalone therapeutic activity	Preclinical	Mechanism-specific auxiliary delivery systems
Selenium Nanoparticles (SeNPs)	ROS modulation; Anti-migratory effects	BBB penetration via surface charge engineering	Narrow therapeutic window; Potential systemic toxicity	Preclinical	Therapeutic potential limited by a narrow and tightly dose-dependent safety margin
Boron-containing Nanoparticles (BNCT agents)	Neutron capture-induced cytotoxicity	Highly localized radiation damage	Requires neutron source; Complex clinical infrastructure	Clinical/Preclinical	Highly effective but infrastructure-dependent clinical strategy

## Data Availability

No new data were created or analyzed in this study. Data sharing is not applicable to this article.
